# Correction: Transcriptomic changes behind *Sparus aurata* hepatic response to different aquaculture challenges: An RNA-seq study and multiomics integration

**DOI:** 10.1371/journal.pone.0327827

**Published:** 2025-07-07

**Authors:** Cláudia Raposo de Magalhães, Kenneth Sandoval, Ferenc Kagan, Grace McCormack, Denise Schrama, Raquel Carrilho, Ana Paula Farinha, Marco Cerqueira, Pedro M. Rodrigues

The Data Availability statement for this article is incorrect. The correct statement is: RNA-seq data for this article have been deposited on the ArrayExpress database (http://www.ebi.ac.uk/arrayexpress) under accession number E-MTAB-12842, available at the link: https://www.ebi.ac.uk/biostudies/ArrayExpress/studies/E-MTAB-12842.
