# Supplementary figures and images for: Transcriptomic changes behind Sparus aurata hepatic response to different aquaculture challenges: An RNA-seq study and multiomics integration
Source: PLoS One. 2024 Mar 22;19(3):e0300472. doi: 10.1371/journal.pone.0300472 (PMC10959376; doi:10.1371/journal.pone.0300472)

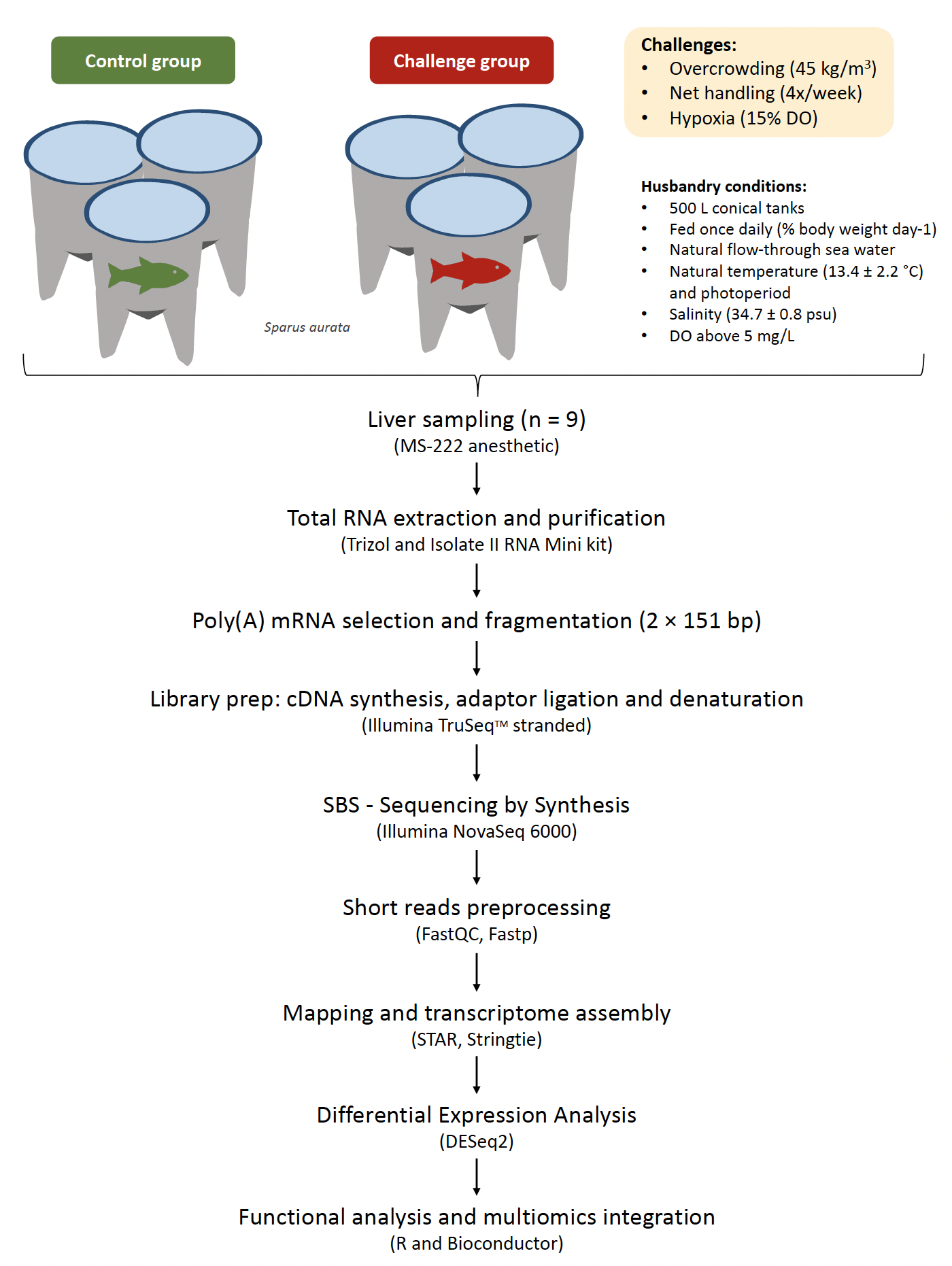

Supplement: S1 Fig — Schematic workflow of the experimental trials and transcriptomics analysis. (TIF) [file pone.0300472.s001.tif]
